# Insecticide Resistance Dynamics and Gene Screening in *Bactrocera dorsalis* (Hendel) Under Varying Selection Pressures

**DOI:** 10.3390/insects17080784

**Published:** 2026-07-29

**Authors:** Doudou Li, Qi Yao, Jianjun Jiang, Yang Yang, Daifeng Cheng, Ling Zeng, Guangwen Liang, Yongyue Lu

**Affiliations:** 1 Department of Entomology, College of Plant Protection, South China Agricultural University, Guangzhou 510642, China; 2 Institute of Plant Protection, Hainan Academy of Agricultural Sciences, Haikou 570100, China; 3 College of Agricultural Engineering, Guangxi Vocational University of Agriculture, Nanning 530003, China

**Keywords:** *Bactrocera dorsalis*, selection pressure, insecticide resistance, trichlorfon, alphamethrin, suppression

## Abstract

Chemical control is the main method used to manage the oriental fruit fly (*Bactrocera dorsalis*) in orchards. However, the oriental fruit fly has gradually developed resistance to multiple insecticides, posing significant challenges for pest resistance management. This study investigated changes in insecticide resistance in oriental fruit flies under different selection pressures. We identified resistance-related genes and their mutation sites using cDNA probes. Our results showed that resistance to trichlorfon decreased significantly in the absence of selection pressure. However, resistance increased when selection pressures were applied every 30 or 60 days. Although the resistance ratio showed a decreasing trend at every 90 and 120 days, a risk of a rebound remained. A large number of homologous sequences were identified in the suppression subtractive hybridization library of insecticide-resistant strains. Resistance to trichlorfon and alphamethrin primarily involves detoxification and metabolic genes, which are often associated with cross-resistance. A single gene may detoxify multiple pesticides, leading to cross-resistance. The four acetylcholinesterase mutations identified in this study are consistent with previous research findings. Moreover, resistance results from the combined action of multiple genes. Based on these findings, this study recommends avoiding continuous use of trichlorfon and maintaining an interval of more than 80 days between applications. It also emphasizes the importance of incorporating pesticide application intervals into integrated pest management strategies. This study provides a theoretical foundation for delaying resistance development in the oriental fruit fly, offers guidance on proper pesticide application intervals in the field, and contributes practical insights for pest resistance management.

## Simple Summary

Despite extensive monitoring data on pest resistance, no specific insecticide application intervals have been proposed to mitigate the development of resistance in agricultural production practices. Research on the relationship between the development of insecticide resistance in oriental fruit flies and the frequency of chemical insecticide use has been poorly reported. This paper provides a theoretical basis for determining insecticide application intervals to prevent resistance development and to maintain or reduce resistance levels in pest management. SSH libraries were constructed, and cDNA technology was used to screen for differentially expressed genes and resistance-related genes. This study plays a role in advancing the theoretical progress in this domain, but also lays the groundwork for further exploration of the molecular mechanisms behind resistance in oriental fruit flies, providing assistance for the sustainable management of pests. It further contributes to a comprehensive understanding of pest resistance mechanisms, aiding in the development of effective control strategies. These findings offer crucial theoretical support and scientific evidence for future research and practical applications.

## 1. Introduction

The oriental fruit fly, *Bactrocera dorsalis* (Hendel), is a significant pest affecting fruits and vegetables in more than 120 countries and regions, mainly locating in Asia, Africa, and Oceania, posing heavy challenges for effective control [1,2,3]. The use of chemical insecticides has significantly changed pest management practices, but over-reliance on pesticides has resulted in increased resistance among the pest populations [4]. Monitoring data show that resistance to chemicals like alphamethrin, trichlorfon, and abamectin in the oriental fruit fly increased from 2003 to 2007 in southern China, with provinces such as Guangdong, Guangxi, Hainan, Yunnan, and Fujian showing the highest levels [5]. Our ongoing surveillance reveals a continuous rise in resistance across 11 populations, with resistance to certain insecticides increasing rapidly between 2010 and 2013, and continuing to grow through 2023 [6]. Over the years, research has improved our understanding of the mechanisms behind resistance in this species, revealing that resistance to malathion is inherited in a polygenic and incompletely dominant manner [7,8,9,10,11,12,13,14]. The development of this resistance is associated with the enhanced activity of detoxification enzymes [15]. Studies have also shown that various substances, including lactic acid bacteria and plant metabolites, play roles in regulating the resistance mechanisms of the oriental fruit fly [16,17,18].

The application of suppression subtraction hybridization (SSH) libraries is widely used for gene screening in areas like plant seed development and microbial gene identification [19,20,21,22,23]. Thamilarasi et al. [24] elucidated the relationship between the expression levels of acyl-CoA δ desaturase in IDS (isopentyl diphosphate synthase)-type decenediphosphate synthase and desaturase genes and resin biosynthesis in lac insects. cDNA microarrays, a type of chip-based technology, enable the assessment of gene expression levels across diverse samples with a shared genetic background [25]. Compared to traditional methods for mRNA expression level detection, cDNA microarrays offer the advantage of high throughput and are increasingly utilized in gene expression analysis [26]. The integration of SSH with cDNA microarray technology represents an effective approach for rapid and high-throughput screening and identification of target genes [27,28]. The use of cDNA microarrays for RNA abundance analysis aims to investigate nutrition–gene interactions and identify genes responsive to dietary (P)-phosphorus, while also confirming the expression variances of phosphorus-responsive genes in feed with varying phosphorus levels [29].

Prolonged exposure to external factors like pesticides and synergists induces various mutations, including single-base substitutions, small DNA insertions or deletions, amino acid substitutions, fusion genes (from chromosomal translocations), and mutations at splicing sites, promoters, or regulatory regions [30,31,32,33]. The studies confirm that mutations at amino acid sites are a fundamental mechanism of insecticide resistance, with minor structural changes enhancing resistance [34,35]. In *Drosophila melanogaster*, an A301S mutation in RDL confers resistance to non-target mushroom compounds (pirocarb and atropine) [36]. Blocking the in vivo interaction between fluralaner and the insect GABA receptor at the N318L site and a mutation in the TMD3 domain of the *Drosophila melanogaster* GABA channel lead to sensitivity to fluralaner [37,38]. The A387G mutation and the overexpression of CYP6CM1 synergistically enhance the resistance of *Bemisia tabaci* to multiple neonicotinoid insecticides [39]. Pan et al. identified an allele in *Drosophila melanogaster* highly similar to CYP6D1, featuring a 15-base pair insertion in its 5′ promoter region. This insertion formed a novel P450 gene, and mutations in this gene were also detected in other countries [40]. The Cyp6a23 mutation is key to the cypermethrin resistance variant in *Drosophila melanogaster* [41]. In *Spodoptera exigua*-resistant strains, a cis-mutation in the promoter region of CYP321B1 generated a novel binding site that enhances transcription factor recruitment, thereby further amplifying expression of this P450 gene [42]. Numerous studies from diverse sources have documented point mutations in the ace gene associated with pesticide resistance in insects. These mutations alter the amino acid sequence, thereby changing the enzyme’s structure and its affinity for substrates and insecticides. Ultimately, this leads to reduced sensitivity to insecticides and the development of resistance [43,44,45]. Sequencing of the ace gene encoding acetylcholinesterase (AchE) in fruit flies worldwide reveals four widespread mutations (I161V, G265A, F330Y, and G368A) in natural populations [46]. A correlation exists between the frequency of the spinetoram resistance allele nicotinic acetylcholine receptor (nAChR) α6 mutation (G275A) in *Drosophila melanogaster* and its use in vineyards [47]. The natural nAChR D*β*1 was replaced with BT*β*1 A58T&R79E, resulting in significantly higher resistance to neonicotinoid insecticides in whiteflies compared to fruit flies; however, replacing the D*β*1 with the wild-type BT*β*1 sequence demonstrated the causal role of the mutation in resistance development [48]. Although there have been a lot of monitoring data showing that there are different degrees of resistance in various regions of China, many studies also confirm that the resistance is related to a variety of substances [49,50,51].

## 2. Materials and Methods

### 2.1. Insect Strains and Insecticides

The susceptible strain of the oriental fruit fly was collected in 2010 from Yangtao Park (23.118722 N, 113.420326 E) in Guangzhou, Guangdong Province, China, and was reared on artificial feed for more than 48 generations without exposure to any pesticides.

The lethal medium concentration for sensitive strain adults was used to treat 52 generations of the oriental fruit fly through population selection. Eventually, a high-resistance strain of the oriental fruit fly to alphamethrin with a resistance ratio of 160.266-fold was obtained. After 32 generations of selection, a medium-resistance strain of the oriental fruit fly against alphamethrin with a resistance ratio of 16.151 was obtained. After 8 generations of selection, a low-resistance strain of the oriental fruit fly against alphamethrin with a resistance ratio of 6.761-fold was obtained (Table 1).

The lethal medium concentration for sensitive strain adults was used to treat 52 generations of the oriental fruit fly through population selection. Eventually, a high-resistance strain of the oriental fruit fly against trichlorfon with a resistance ratio of 52.679-fold was obtained. After 22 generations of selection, a medium-resistance strain of the oriental fruit fly to trichlorfon with a resistance ratio of 15.611-fold was obtained. After 8 generations of selection, a low-resistance strain of the oriental fruit fly to trichlorfon with a resistance ratio of 5.377-fold was obtained (Table 1).

The breeding and resistance selection environment was maintained at a constant temperature (25–28 °C), constant humidity (60 ± 10%), and a 16:8 light/dark cycle. Trichlorfon (purity ≥ 90%, CAS: 52-68-6) was produced by Jiangsu Nantong Agrochemical Co. Ltd., Nantong, Jiangsu, China; alphamethrin (purity ≥ 95%, CAS: 65731-84-2) was produced by Guangdong Liwei Chemical Industry Co. Ltd., Guangzhou, Guangdong, China; and acetone, analytically pure A.R., was produced by Guangzhou Chemical Reagent Factory, Guangzhou, Guangdong, China.

### 2.2. Bioassay and Selection

Bioassays were conducted using the residual contact method [37]. Stock solutions of insecticides were diluted to six concentration gradients. Each concentration (5 mL) was placed into a 250 mL flask to form a uniform film, and 20 adult flies (sex ratio 1:1, 3–5 days old) were added to each flask. After sealing with gauze and providing a honey-water feeder, mortality was recorded after 24 h. The experiment was validated if mortality in the control group was below 10%. Each concentration had six replicates.

### 2.3. Construction of the SSH Library

Eight adult individuals (5 days post-eclosion, with a 1:1 sex ratio) were selected from the resistant and susceptible strains of *Bactrocera dorsalis* to construct the SSH libraries for the susceptible and resistant strains. Total RNA was extracted using the Trizol (Invitrogen, Guangzhou Genewindows Biotech Ltd., Guangzhou, Guangdong, China) method. First-strand cDNA synthesis, ds cDNA synthesis and purification, restriction enzyme digestion and purification of ds cDNA with Rsa I were performed. The following kits were used: SMARTer PCR cDNA Synthesis Kit (Cat. No. 634925), Advantage 2 PCR Kit (Cat. No. 639206), Advantage cDNA PCR Kit (Cat. No. 634105), and PCR-Select cDNA Subtraction Kit (Cat. No. 637401), and they all were purchased from Clontech Laboratories, Inc., Mountain View, CA, USA, and all operations were performed strictly according to the instructions.

Hybridization: After the junction connection efficiency test was completed, excessive driver cDNA and tester cDNA are used for the first reduction hybridization, and the differentially expressed genes are enriched after hybridization. The two reaction products after the first hybridization are mixed together, and newly denatured driver cDNA is added for re-hybridization, which can further enrich the differentially expressed genes.

The following kits were used for PCR amplification, PCR product purification, and subtractive cDNA library cloning: PCR amplification kit (Cat. No. R011), PCR Product Purification Kit (Cat. No. 28104, Qiagen, Hilden, Germany), DH5α competent cells, and PMD20-T vector, purchased from Takara Bio, Mountain View, USA. All operations were performed strictly according to the instructions.

The specific sequence used to detect junction connection efficiency in this study was the skeletal muscle gene of the oriental fruit fly (Table S1). This study employed PCR methods to compare the abundance of the housekeeping gene G6PDH in the library before and after subtractive hybridization to assess the effects of subtractive hybridization on detection.

### 2.4. cDNA Gene Chip Hybridization

Extraction of SSH library recombinant plasmids, PCR amplification and purification of recombinant plasmids, spot preparation of cDNA microarrays, fluorescent labeling and hybridization of probe RNA samples, and chip scanning (not sure how this list of actions appears here before sentences): Recombinant plasmid extraction was performed using the SSH library recombinant plasmid extraction kit. PCR amplification was conducted using the adapter nested primers Nest PCR Primer 1 and Nest PCR Primer 2R from the SSH kit. The amplified products were purified using an isopropanol/ethanol precipitation method. The spot preparation of purified PCR products and internal control gene fragments (Table S2) and chip spot preparation were both outsourced to Beijing Bio-Ocean Biotechnology Co. Ltd. Fluorescent labeling of RNA samples was performed using the Jingxin^®^ cRNA Amplification and Labeling Kit. Chip hybridization was outsourced to Beijing Bio-Ocean Biotechnology Co. Ltd. (Beijing, China) Chip scanning was performed using the LuxScan 10KA dual-channel laser scanner (CapitalBio Company, Beijing, China).

### 2.5. Preparation of Gene Chips for Monitoring Resistance in the Oriental Fruit Flies

Using the resistance-related differential genes identified through SSH and cDNA chip separation, the trichlorfon and alphamethrin strains were selected as target genes for monitoring resistance (NCBI accession number PZ465964-PZ465970), the SSH library recombinant plasmid was used as the template, and Nester PCR Primer 1 and Nester PCR Primer 2R were used as amplification primers to amplify the gene sequences of the resistance-related differential unigenes. *β*-actin and 18S genes were used as internal control genes (Table S3). The purification of target genes and internal reference genes, spot preparation, chip spot preparation, fluorescent labeling of RNA samples (CrystalCore^®^ cRNA Amplification Labeling Kit), and chip hybridization were all outsourced to Beijing Bio-Ocean Bio-Technology Co. Ltd. The chip was scanned using a LuxScan 10KA dual-channel laser scanner (CapitalBio). Chip images and data were then acquired.

### 2.6. Cloning of the Ace Gene

After sequencing the target fragment, the sequences were assembled using Mega5.0 software and subsequently translated into amino acid sequences. The amino acid sequences of the ace gene (Table S1) from different strains were compared with those of the sensitive strain as a reference.

### 2.7. Data Analysis

Using SPSS version 20.0 (SPSS, Inc., IBM, Armonk, NY, USA) statistical software, data were processed to calculate toxicity regression equations, the median lethal concentration (LC_50_), 95% confidence intervals (95% CLs), and correlation coefficients (R^2^) [5]. Resistance levels were classified based on the resistance ratio (LC_50_ of resistant strains/LC_50_ of susceptible strains) as follows: resistance ratio < 3 indicates susceptible level; 3–10-fold indicates low resistance level; 10.1–40-fold indicates medium resistance level; 40.1–160-fold indicates high resistance level; >160-fold indicates extremely high resistance level [52].

Blast2GO version 5.2 (BioBam Bioinformatics, Valencia, Spain), MEGA version 11.0 (Biodesign Institute, Tempe, AZ, USA), and the National Center for Biotechnology Information (NCBI) web database (http://blast.ncbi.nlm.nih.gov/Blast.cgi, URL accessed on 3 December 2012) were used for annotation comparison to screen out the differentially expressed genes in the SSH library. Relative quantitative data analysis was performed using the 2^−△△CT^ method, and data processing was done using REST version 2009 (Qiagen, Hilden, Germany).

## 3. Results

### 3.1. Changes in Resistance of the Medium-Resistant Strain of the Oriental Fruit Fly to Trichlorfon Under Different Selection Intervals

Trichlorfon is commonly used in the field to control the oriental fruit fly, *Bactrocera dorsalis*. Different resistance levels to trichlorfon have already been reported in field populations. Therefore, this study aimed to evaluate the toxicity of trichlorfon to the oriental fruit fly and to determine how long pesticide-free maintenance or different selection intervals are needed to reduce or avoid further increases in resistance. A moderately trichlorfon-resistant strain was selected using the residual film method and used as the research material. This strain was then subjected to selection at different time intervals. The results may provide a basis for the rational use of trichlorfon in the field control of B. dorsalis. Under pesticide-free conditions, the resistance level of this moderately resistant strain decreased after 271 days, with LC_50_ falling from the initial value of 45.312 mg/L to 30.547 mg/L, and the resistance decreased from an initial 12.961-fold to 8.738-fold (Table 2).

At a selection frequency of every 30 days, the LC_50_ for the adult oriental fruit fly strain with medium trichlorfon-resistant increased from 45.312 mg/L to 70.592 mg/L, with the resistance ratio rising from 12.961-fold to 20.192-fold (Table 3). Under the condition of selecting once every 60 days using trichlorfon, the LC_50_ of the medium trichlorfon-resistant oriental fruit fly continuously increased, and the resistance ratio increased from 12.961-fold to 16.250-fold (Table 4). Under the selection condition of once every 90 days, the LC_50_ of the medium trichlorfon-resistant strain of the oriental fruit fly showed a slow decrease, indicating that after developing medium resistance to trichlorfon, using trichlorfon once every 90 days does not further increase resistance (Table 5). After selection every 120 days, no notable reduction in the toxicity level of the trichlorfon-resistant oriental fruit fly strain was observed (Table 6).

### 3.2. Quantitative Relationship Between Resistance Trends and Treatment Frequency in the Medium-Resistant Strain Against Trichlorfon of the Oriental Fruit Fly

**Figure 1:**
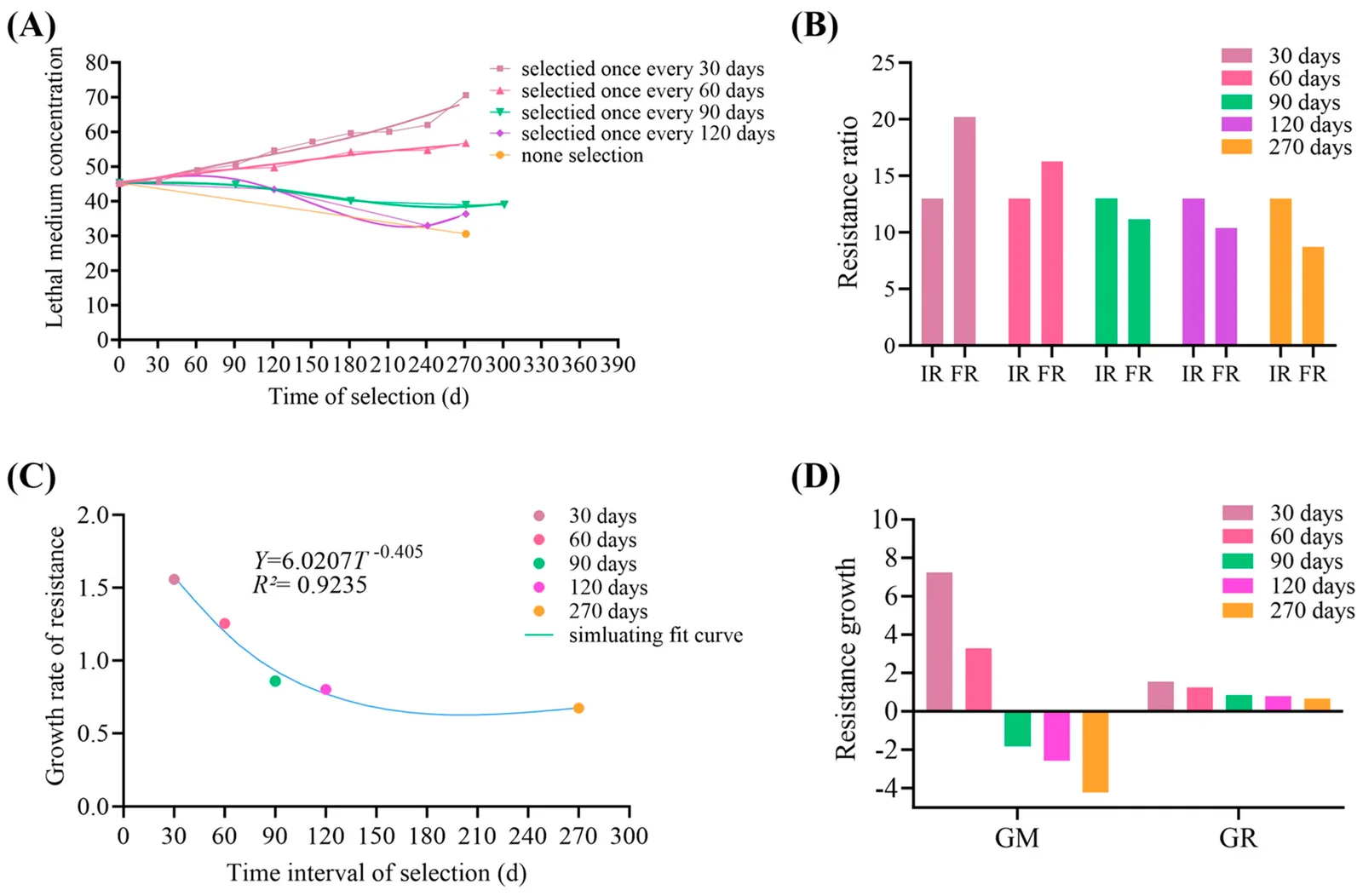
The effect of different selection intervals on *Bactrocera dorsalis* strains resistant to trichlorfon: (**A**) Changes in LC_50_ concentration, (**B**) Changes in resistance ratio, (**C**) Resistance growth rates at different time intervals, and (**D**) Changes in resistance growth. Abbreviations: IR, initial resistance ratio; FR, final resistance ratio; GM, growth multiple of resistance; GR, growth rate of resistance.

The trend in resistance development of the oriental fruit fly varied under different selection treatment conditions (Figure 1). The resistance growth multiples of the oriental fruit fly strain resistant to trichlorfon were 7.231, 3.289, −1.831, −2.567, and −4.223-fold under selection intervals of 30, 60, 90, 120, and 270 days, respectively. The resistance growth rates showed a decreasing trend with values of 1.558, 1.254, 0.859, 0.802, and 0.674, respectively (Table S4). The resistance growth multiples and growth rate showed a gradual decreasing trend (Figure 1A,B,D). A model was fitted to the selection interval time and resistance growth rate. The results of the selection experiment with different intervals showed that a reasonable interval should be maintained when the same insecticide is applied repeatedly. Based on our results, an interval of more than 84 days is recommended. When trichlorfon was applied twice at this interval, the resistance level of *Bactrocera dorsalis* did not increase (Figure 1C).

### 3.3. Differentially Expressed Genes in SSH Library of Trichlorfon- and Alphamethrin-Resistant Strains

**Figure 2:**
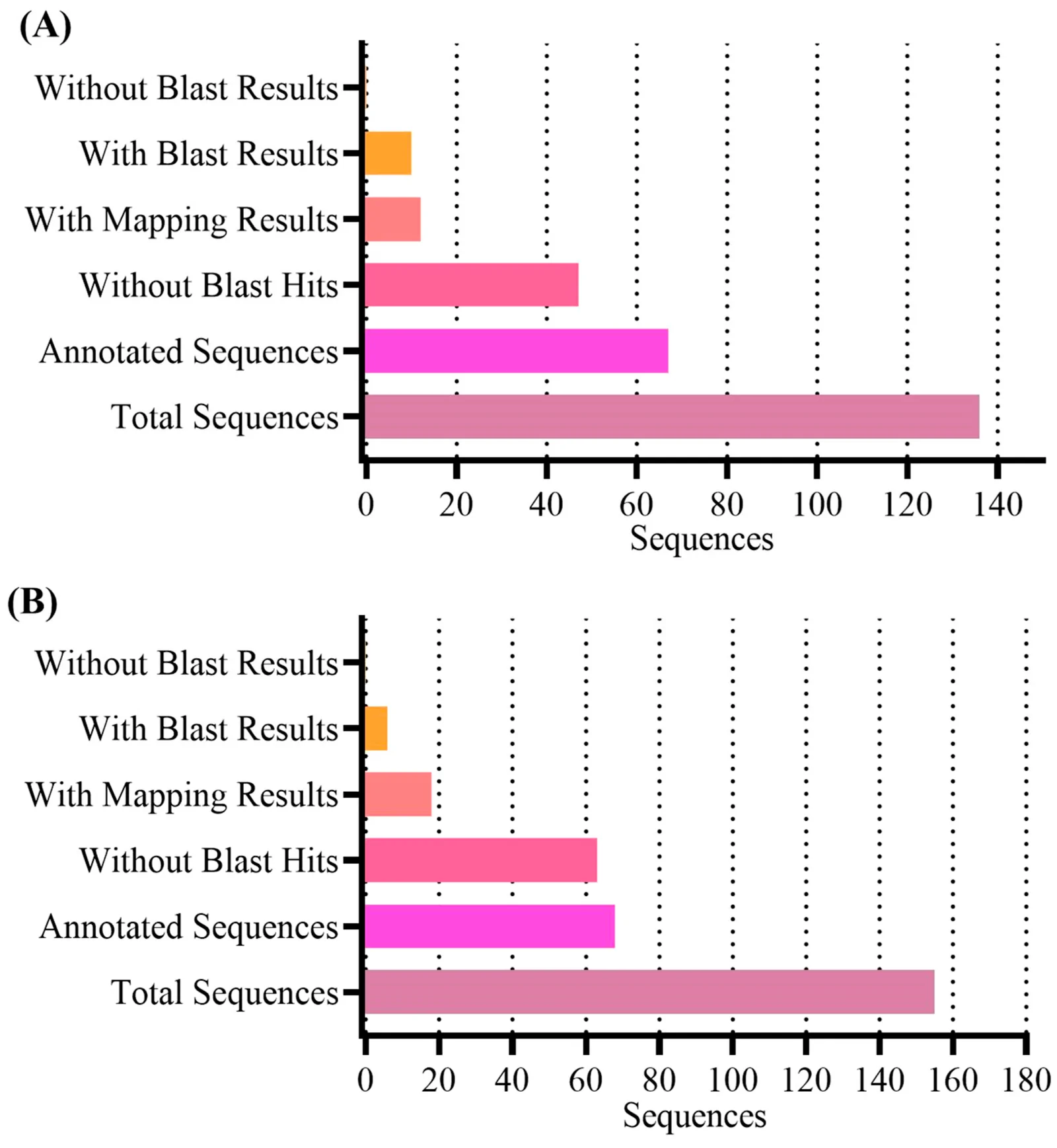
Distribution of cDNA libraries of oriental fruit fly-resistant strains: (**A**) Trichlorphon strain, (**B**) Alphamethrin strain.

A total of 391 and 311 differentially expressed genes were obtained from the SSH library of the oriental fruit fly-resistant strain through cDNA chip screening and sequencing, and these genes were clustered. This resulted in 136 (trichlorphon (in the heading of this paragraph: “trichlorfon” but here is “trichlorphon”) strain) and 152 (alphamethrin strain), each of which was greater than 100 bp (Table S5). Homologous BLAST alignment with the NCBI Nucleotide Database (Nt) revealed that the trichlorphon-resistant strain had 89 homologous sequences, accounting for 65.44%, and the alphamethrin-resistant strain had 92 homologous sequences, accounting for 60.53% (Figure 2).

According to the molecular functional annotation of the cDNA library of the oriental fruit fly strain resistant to trichlorphon and alphamethrin, the differentially expressed genes associated with resistance in the oriental fruit fly are primarily functional genes related to oxidoreductase activity, hydrolase activity, transferase activity, electron carrier activity, structural molecule activity, ion binding, protein binding, small molecule binding, and nucleic acid binding (Tables S6 and S7). P450 family genes are highly expressed in the library. Among the differentially expressed genes in the trichlorphon-resistant strain, there is also one juvenile hormone esterase, one glutathione *S*-transferase gene, and one multidrug resistance gene.

### 3.4. Quantitative PCR Validation of Differentially Expressed Genes in Trichlorfon- and Alphamethrin-Resistant Strains

**Figure 3:**
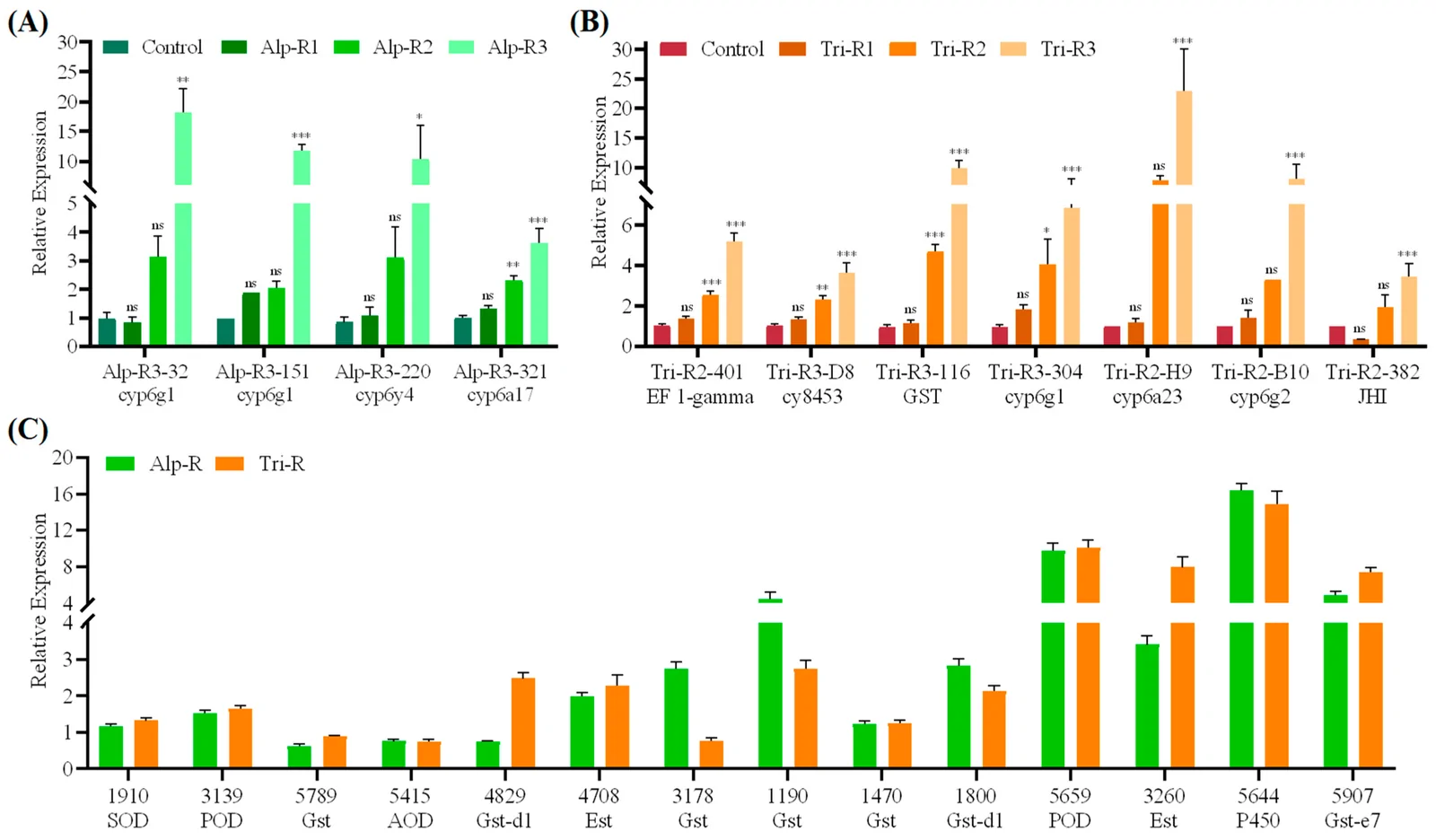
Relative expression levels of differentially expressed genes in SSH libraries from resistance strains of *Bactrocera dorsalis*: (**A**) Alphamethrin-resistant strains, (**B**) Trichlorfon-resistant strains, (**C**) Resistance-related genes. Alp-R3-32: Alp-R3 denotes the strain, 32 is a cloned spot; 1910 SOD: 1910 denotes sequence clone number, SOD represents the gene. JHI: juvenile hormone-inducible; EF-gamma: elongation factor 1-gamma. *** *p* < 0.001, ** *p* < 0.01, * *p* < 0.05, ns indicates *p* > 0.05.

Among all strains, the highly resistant strain showed the highest relative upregulation of differentially expressed genes (Figure 3A,B). All control groups were normalized to 1 to allow relative comparison with the experimental groups, and the expression levels of all differentially expressed genes were higher in the highly resistant and moderately resistant strains than in the sensitive strain (Table S8). Nine genes showed a greater than 2-fold increase in expression levels in both strains (Table S9). Fourteen homologous genes from the P450 and GST gene families, as well as non-specific esterases, were selected from the SSH library of oriental fruit fly-resistant strains (Figure 3C). Compared with the susceptible strain, among the 14 screened genes, 8 genes showed more than a 2-fold increase in expression in the Alp-R3 strain, and 8 (not sure what this: 8 genes or 8-fold) in the Tri-R3 strain. Six genes (GST-1190/1800/5907, POD-5659, Est-3260, and P450-5644) showed more than a 2-fold upregulation in both strains.

**Figure 4:**
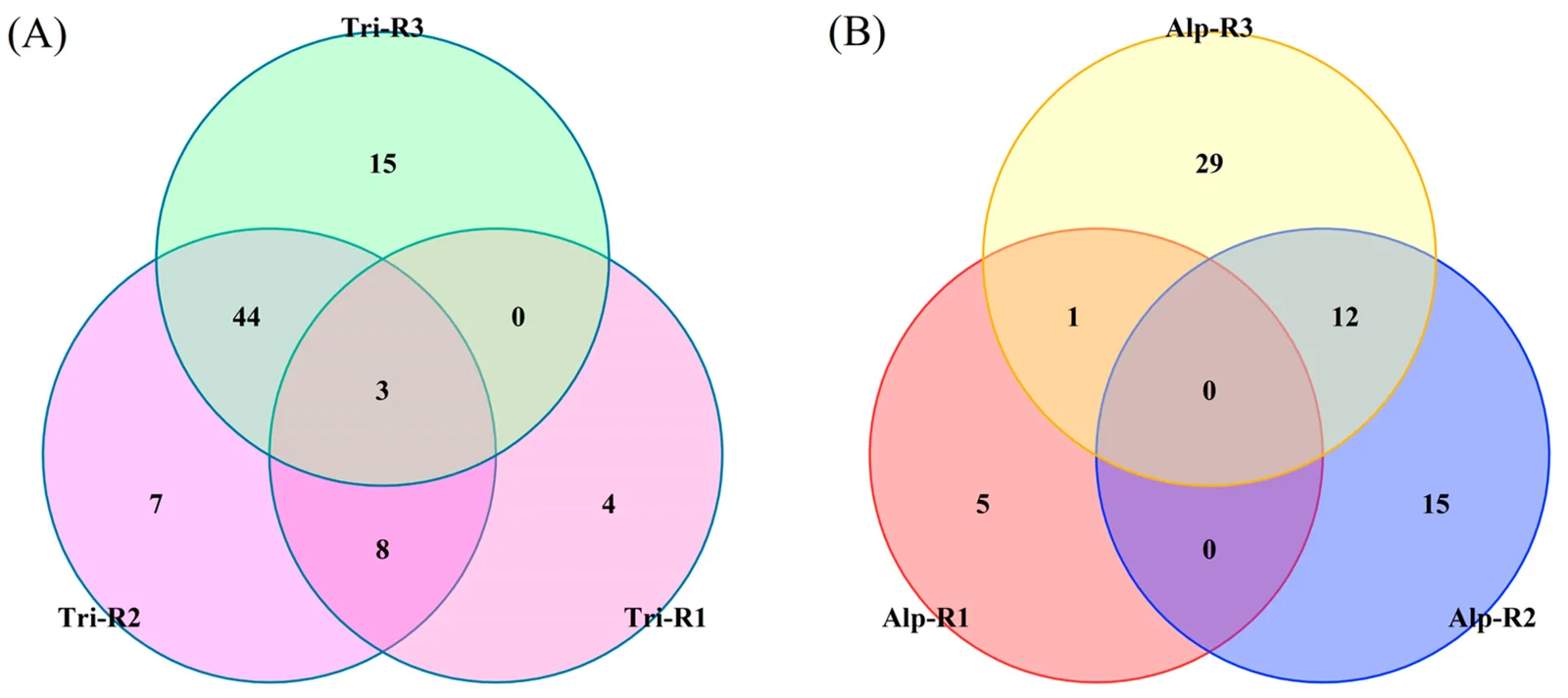
Expression of resistance samples in the microarray: (**A**) trichlorphon-resistant strains; (**B**) alphamethrin-resistant strains. Annotation: The numbers in each circle represent the number of genes with a signal ratio ≥2-fold in the chip for each test sample.

Based on the results from the SSH library and cDNA chip analysis, a subset of unigenes was selected from two types of insecticide-resistant strains as target genes for resistance monitoring (Table S10): Tri-R1, Tri-R2, and Tri-R3 had 4, 15, and 15 upregulated genes, respectively, in the target gene monitoring chip (Figure 4A); Alp-R1, Alp-R2, and Alp-R3 had 5, 15, and 27 upregulated genes, respectively, in the target gene monitoring target gene chip (Figure 4B). The prepared target-specific gene chips effectively distinguished between different resistance strains, and the number of upregulated genes reflected their resistance status.

### 3.5. Analysis of Ace Gene Cloning and Mutations in Pesticide-Resistant Oriental Fruit Fly Strains

To further clarify whether target-site variation in AChE (there are two versions of this in this article: AchE and AChE) was involved in resistance among the resistant strains, the full-length ace sequences were amplified, sequenced, and compared among different strains of *Bactrocera dorsalis*. In addition, the conserved functional sites of AChE were examined by comparison with those of *Drosophila melanogaster*. This analysis allowed us to determine whether mutations occurred in conserved catalytic regions or in other amino acid positions of the ace sequence. Sequencing and assembly yielded the full-length *ace* sequences (2022 bp) for various strains of the oriental fruit fly, which exhibited over 99% sequence similarity to previously reported *ace* sequences of this species. A comparison of the AChE amino acid sequences between *Bactrocera dorsalis* and *Drosophila melanogaster* revealed that the signal peptide of the oriental fruit fly sequence is 55 amino acids longer than that of *Drosophila melanogaster* (38aa). Compared to the conserved amino acid sites for enzyme activity in *Drosophila melanogaster* (S245, E364, and H477), these three sites in the oriental fruit fly are also highly conserved, with no mutations observed at the base level. Four amino acid mutations were identified in the three resistant strains: Tri-R2, Tri-R3, and Alp-R3, namely I214V, G488S, Q643R, and G420A (Table 7). These four mutation sites correspond to the ACE mutation sites reported by Jiang et al. [53].

## 4. Conclusions and Discussion

Since the production and widespread application of organic synthetic insecticides, pest resistance has continuously arisen and become a significant issue in pest control practice. Pest control has gradually evolved, shifting from chemical pesticides with quick results to greener, pollution-free biological and agricultural control [54,55,56,57]. It was observed that under different selection intervals, when selection occurred every 30 or 60 days, the resistance level of oriental fruit flies to trichlorfon continued to increase. This increase became more pronounced with each subsequent selected generation. The longer the selection interval, the slower the development of resistance. When the selection interval was extended to 90 days or longer, the resistance level no longer increased and instead tended to decline as the interval lengthened. Based on the power function model, continuous field application of insecticides with intervals exceeding 84 days could prevent the development of resistance. The results of this experiment are consistent with those of Yao et al. [58].

Insects develop pesticide resistance for many reasons [59,60,61,62,63]. Differences between resistant and sensitive strains include epidermal stratum corneum thickness and color, fat body structure, chitin-layer lamellar structure, cell spacing, microsatellite DNA polymorphism, detoxification enzyme activity, protein response rate, protein involvement, and intestinal symbiotic bacteria [64,65,66]. Several methods are available for screening resistance genes, including RNA sequencing, transcriptome sequencing, SSH library, and cDNA library [67,68]. In the SSH libraries of the Tri-R and Alp-R strains, 79 and 86 sequences, respectively, had homologous sequences with known functions. These genes have been confirmed to be related to the insecticide resistance mechanism. SSH library annotation and quantitative analysis revealed that upregulation of gene expression was positively correlated with the insecticide resistance level. Both pesticide resistance-related genes and differentially expressed genes were upregulated by more than 2-fold in the Alp-R3 and Tri-R3 strains. This suggests that the involvement of the same detoxification metabolic gene in the detoxification metabolic activities of different target types of insecticides may be one of the main reasons for the cross-resistance of insects.

AchE persists globally at moderate frequencies in insecticide-resistant alleles and exhibits a reversal of beneficial advantage, stemming from the dominance and adaptive stability of resistant *ace* alleles [69,70,71]. The acetylcholinesterase 1 G119S substitution confers insecticide resistance in *Culex pipiens mosquitoes* [72]. Three-point mutations, I199V, G303A, and F368Y, significantly enhance *Drosophila melanogaster* resistance to the organophosphorus pesticide malathion [73]. Global population genomic data revealed that three missense variants in ACE, T3I, G303A, and F368Y, were found to exhibit significantly elevated frequencies in high-altitude populations, such as those in North China and Qinghai–Tibet, but these mutations have undergone positive selection in the local environment [74]. Allele-driven technology restored the wild-type *ace* allele and successfully eliminated pre-existing malathion-resistant alleles in the laboratory, demonstrating the potential of gene drives to reverse resistance caused by *ace* mutations [75]. Mutations such as G328A, I159V, G433S, and G365A are the primary causes of target insensitivity to organophosphate pesticides, including malathion and chlorpyrifos [76]. The four AchE mutations identified in this study are consistent with those reported by Jiang et al. [53]. These mutations may also contribute to the increased resistance of the oriental fruit fly to trichlorfon and alphamethrin [77]. Although AchE is not the primary target of pyrethroid insecticides, Ace-1 280S mutations have also been detected in *Anopheles gambiae* s.l. populations showing high resistance to pyrethroids [78]. This finding is consistent with our observation that four mutation sites in the AchE gene were detected in the alphamethrin-resistant strain. However, because AchE is not the primary target of pyrethroid insecticides, these mutations should be interpreted as part of the complex resistance background rather than direct evidence that AchE mutations confer alphamethrin resistance.

Considering economic benefits, the concept of Integrated Pest Management (IPM) and Integrated Pest Control (IPC) should be observed. The “prevention first, integrated control” should guide pest prevention and control, and the pests that have already developed resistance should be effectively treated [79,80]. For resistant pests, insecticides with different modes of action should be rotated to avoid repeated exposure to insecticides with the same mode of action across generations. Many studies have shown that rotating insecticides with different modes of action can effectively reduce pest resistance, and the importance of insecticide rotation is well-established [81,82,83]. The results have laid a theoretical foundation for the green pest control technology and development in agricultural pest management, and provided guidance for exploring new avenues for pest control. However, this study has some limitations. First, the experimental insects were laboratory strain and experimental conditions differ significantly from field conditions. Therefore, the results primarily offer a reference for using trichlorfon to control oriental fruit flies with specific application intervals, and further research on rotating insecticides with different modes of action is needed. Second, while the resistance genes identified in this study provide valuable insights, the techniques used have potential limitations, and further research is needed.

## Figures and Tables

**Table 1:** Resistance levels of *Bactrocera dorsalis* adults to alphamethrin and trichlorfon.

Strains	Insecticides	Regression Equation	LC_50_ (95% CL; mg/L)	R^2^	RR	χ^2^	*p*
SS	Trichlorfon	y = −1.466 + 2.697x	3.496 (3.076–3.963)	0.9721	1.000	7.602	<0.01
SS	Alphamethrin	y = 3.9516 + 1.9919x	3.360 (2.6567–4.2492)	0.9279	1.000	14.504	<0.01
Alp-R1	Alphamethrin	y = −2.1825 + 2.0757x	22.717 (7.8500–64.6000)	0.9794	6.761	3.064	>0.05
Alp-R2	Alphamethrin	y = 1.0020 + 2.3049x	54.269 (47.8818–61.5087)	0.9806	16.151	18.394	<0.01
Alp-R3	Alphamethrin	y = −9.5100 + 8.9742x	538.494 (517.3914–560.4573)	0.9491	160.266	3.295	>0.05
Tri-R1	Trichlorfon	y = −6.110 + 4.796x	18.797 (16.524–20.998)	0.9370	5.377	7.656	<0.01
Tri-R2	Trichlorfon	y = −1.014 + 2.709x	54.576 (46.601–63.679)	0.9877	15.611	21.539	<0.01
Tri-R3	Trichlorfon	y = −3.7213 + 8.0354x	184.167 (169.0725–200.6098)	0.9296	52.679	23.191	<0.01

Annotation: R^2^, Correlation coefficient; RR, resistance ratio; CL, confidence limit; χ^2^, Chi-square. LC_50_ values were considered significantly different if their confidence limits did not overlap. The same below.

**Table 2:** Changes in resistance of trichlorfon-resistant *Bactrocera dorsalis* adults without selection.

No. of Selection	Time of Selection (d)	Regression Equation	LC_50_ (95% CL; mg/L)	R^2^	RR	χ^2^	*p*
1	1	y = −7.264 + 4.386x	45.312 (36.393–55.563)	0.9970	12.961	36.555	<0.01
	271	y = −4.328 + 2.914x	30.547 (23.292–38.889)	0.9985	8.738	8.356	<0.01

**Table 3:** Changes in resistance of trichlorfon-resistant adults of *Bactrocera dorsalis* selected every 30 days (this format also applies to the following three tables).

No. of Selection	Time of Selection (d)	Regression Equation	LC_50_ (95% CL; mg/L)	R^2^	RR	χ^2^	*p*
1	1	y = −7.264 + 4.386x	45.312 (36.393–55.563)	0.9970	12.961	36.555	<0.01
2	31	y = −6.346 + 3.821x	45.819 (33.965–57.795)	0.9664	13.106	29.966	<0.01
3	61	y = −4.466 + 2.644x	48.917 (36.886–63.727)	0.9680	13.992	18.181	<0.01
4	91	y = −8.530 + 5.008x	50.496 (41.984–61.201)	0.9884	14.444	0.905	>0.05
5	121	y = −5.628 + 3.239x	54.620 (42.881–69.607)	0.9985	15.624	29.264	<0.01
6	151	y = −6.254 + 3.559x	57.187 (45.658–71.404)	0.9757	16.358	37.295	<0.01
7	181	y = −7.701 + 4.338x	59.629 (45.898–73.449)	0.9737	17.056	0.762	>0.05
8	211	y = −6.339 + 3.564x	60.057 (48.269–74.965)	0.9788	17.179	29.565	<0.01
9	241	y = −3.513 + 1.960x	61.974 (44.373–87.243)	0.9690	17.727	4.646	>0.05
	271	y = −4.815 + 2.604x	70.592 (52.789–93.618)	0.9930	20.192	20.377	<0.01

**Table 4:** Adults of the trichlorfon-resistant *Bactrocera dorsalis* were selected every 60 days.

No. of Selection	Time of Selection (d)	Regression Equation	LC_50_ (95% CL; mg/L)	R^2^	RR	χ^2^	*p*
1	1	y = −7.264 + 4.386x	45.312 (36.393–55.563)	0.9970	12.961	36.555	<0.01
2	61	y = −10.484 + 6.212x	48.724 (39.573–58.381)	0.9925	13.937	1.211	>0.05
3	121	y = −4.737 + 2.792x	49.736 (37.005–64.821)	0.9803	14.227	18.140	<0.01
4	181	y = −7.039 + 4.059x	54.207 (44.167–66.822)	0.9980	15.505	22.942	<0.01
5	241	y = −5.259 + 3.025x	54.769 (42.896–69.223)	0.9706	15.666	26.667	<0.01
	271	y = −6.513 + 3.712x	56.809 (43.061–71.677)	0.9680	16.250	0.5587	>0.05

**Table 5:** Adults of the trichlorfon-resistant *Bactrocera dorsalis* were selected every 90 days.

No. of Selection	Time of Selection (d)	Regression Equation	LC_50_ (95% CL; mg/L)	R^2^	RR	χ^2^	*p*
1	1	y = −7.264 + 4.386x	45.312 (36.393–55.563)	0.9970	12.961	36.555	<0.01
2	91	y = −4.872 + 2.950x	44.827 (32.669–58.002)	0.9975	12.822	18.694	<0.01
3	181	y = −5.167 + 3.224x	40.068 (31.694–50.044)	0.9654	11.461	17.963	<0.01
4	271	y = −3.157 + 1.986x	38.911 (25.282–54.127)	0.9618	11.130	6.792	<0.01
	301	y = −4.671 + 2.936x	39.008 (29.819–49.987)	0.9783	11.158	14.983	<0.01

**Table 6:** Adults of the trichlorfon-resistant *Bactrocera dorsalis* were selected every 120 days.

No. of Selection	Time of Selection (d)	Regression Equation	LC_50_ (95% CL; mg/L)	R^2^	RR	χ^2^	*p*
1	1	y = −7.264 + 4.386x	45.312 (36.393–55.563)	0.9970	12.961	36.555	<0.01
2	121	y = −4.763 + 2.909x	43.387 (32.310–55.605)	0.9975	12.410	7.364	<0.01
3	241	y = −5.133 + 3.381x	32.981 (25.600–41.486)	0.9844	9.434	14.422	<0.01
	271	y = −4.407 + 2.824x	36.339 (27.630–46.660)	0.9960	10.394	10.466	<0.01

**Table 7:** Frequencies of amino acid substitutions in AChE in insecticide-resistant strains of *B. dorsalis*.

Strains	Frequency (%)			
	I159V	G365A	G433S	Q588R
SS	0	0	0	0
Tri-R_2_	100	0	100	100
Tri-R_3_	100	100	100	100
Alp-R_2_	0	0	0	0
Alp-R_3_	100	100	100	100

## Data Availability

The original contributions presented in this study are included in the article/Supplementary Material. Further inquiries can be directed to the corresponding author.

## Supplementary Materials

The following supporting information can be downloaded at https://www.mdpi.com/article/10.3390/insects17080784/s1. Table S1. Gene primer list. Table S2. Primers used in quantitative real-time PCR to detect differentially expressed genes. Table S3. Primers used in quantitative PCR for the cDNA library. Table S4. Growth rate of resistance of B. dorsalis adults to after selection with different time interval. Table S5. Sequence composition table of SSH library contigs from oriental fruit fly resistant strains. Table S6. The classification of molecular function from SSH library of resistant to trichlorphon. Table S7. The classification of molecular function from SSH library of resistant to alphamethrin. Table S8. Differentially expressed genes in resistant strains of oriental fruit fly RT-PCR. Table S9. Relative expression levels of resistance-related genes in insecticide-resistant strains of various types. Table S10. The resistance target gene of the microarray.
