# Supplementary material for: Insecticide Resistance Dynamics and Gene Screening in Bactrocera dorsalis (Hendel) Under Varying Selection Pressures
Source: Insects. 2026 Jul 29;17(8):784. doi: 10.3390/insects17080784 (PMC13513017; doi:10.3390/insects17080784)
Supplement: Supplementary file 1 [file insects-17-00784-s001.zip › insects-4231940-supplementary.pdf]

Table S1. Gene primer list

Gene Name	Sequence (F/R)
G6PDH	5'-GCTGCCGAAACCTACAAA-3'/5'-CGCTTATCATAGCCACCC-3'
β-actin	5'-GATGGTTGGTATGGGACA-3'/5'-GGTGGTGGTGAAAGAGTAA-3'
18S	5'-TAAAGCAGGCTCCAAA-3'/5'-ACGACGGTCCAAGAAT-3'
ace1	5'-CGAGCAGTGTTAATAAGGTG-3'/5'-AAATGCCCGAGTAGGAGT-3'
ace2	5'-AATGCGTCGCTGTTGT-3'/5'-AATGCGTCGCTGTTGT-3'

Table S2. Primers used in quantitative real-time PCR to detect differentially expressed genes

Strains	Gene Name	Sequence (F/R)
SS-B4	unkown	5'-CAAGCCAGCAGTGAAT-3'/5'-CCGTTGGTATAGGAGGT-3'
SS-331	unkown	5'-ACGGATCTTCTTCTTCT-3'/5'-GGGACAAACAGCCAGA-3
Alp-R3-32	cyp6g1	5'-GCGGCAGTTGGTATTT-3'/5'-AGCGTCGTTAGTCTTCG-3'
Alp-R3-151	cyp6g1	5'-TGGTGGCAATCACATCGG-3'/5'-TCGGCACGGAGTTGGAAGC-3'
Alp-R3-175	cyp6g1	5'-TTTCGTTCTCAAATCCTTC-3'/5'-GATAAATACTGCTTGCCTC-3'
Alp-R3-220	cyp6y4	5'-TCAACGCCGAGAATAAGAAA-3'/5'-CGATGCGAGAACCAATACAG-3'
Alp-R3-231	cyp6g4	5'-GGGGAGTGTTATTGTGAA-3'/5'-ACTGTAGCGACACCAAAC-3'
Alp-R3-321	cyp6a17	5'-AGATTCTCCTTCGCACCA-3'/5'-TACTCTTTCAACGCCTCC-3'
Alp-R2-F12	p450	5'-GCGAGCACAAGTCGTCAC-3'/5'-CCGCCAAGAGGTAAGTAGC-3'
Tri-R2-A9	cyp6g1	5'-GGGCTTGTCTATGTTTGGTG -3'/5'-CGCTGGCTTGTTGAGTATGT-3'
Tri-R2-H9	cyp6a23	5'-TGGTGGTTGAAGAGGTTT-3'/5'-AATTGAAGTTATGCGTGAG-3'
Tri-R2-B10	cyp6g2	5'-TTCAGCCTCCTGTTTG-3'/5'-AGTTGACGCCAGTTTT-3'
Tri-R2-382	JHI	5'-ATCTATGAAGTCCGTTGTGG-3'/5'-TGTCGGTATGGTGAGTATTGT-3'
Tri-R2-401	EF 1-gamma	5'-AAATCGCTAAACTCCAACGG-3'/5'-TTCCTCCTCTTCCTCTTCCTCT-3
Tri-R3-D8	cy8453	5'-CTTTCGCAACTGCCTCTT-3'/ 5'-CGTGCCATTGACTTCTTC-3'
Tri-R3-40	cyp6g1	5'-GGGTTGGTCTATGTTTGGTG-3'/5'-TAACGCTGGCTTGTTGAGTA-3'
Tri-R3-116	GST	5'-ATAAGCCTCCAGCACAGT-3'/5'-TGATTCGCTCTATCCAAA-3'
Tri-R3-304	cyp6g1	5'-TATGCGAAGACTAACGAC-3'/5'-GACGGAGTGTAAAGGTGA-3'

Annotation: SS, sensitive strain of oriental fruit fly; Tri-R1/R2/R3, Oriental fruit fly strains with low, medium, and high resistance to trichlorfon; Alp-R1/R2/R3, citrus fruit fly strains with low, medium, and high resistance to alphamethrin; JHI, juvenile hormone-inducible; EF-gamma, elongation factor 1-gamma.

Table S3. Primers used in quantitative PCR for the cDNA library

Sequence clone number	Resistance-associated genes	Sequence (F/R)
1190	Gst	5'-CCAGTCCACCAGTCCGTAG-3'/5'-TCGCCTGACCATCATCCA-3'

1470	Gst	5'-TTCTTCGCTTGTATTCTATG-3'/5'-GCTGCCTCACTACCACCT-3'
1800	Gst-d1	5'-AGCGTGCTGTCGTCAATC-3'/5'-CCGTCCCTCCAGGAATGT-3'
3178	Gst	5'-CTACACTCAGCCCACCAT-3'/5'-GTCCACCAAGTAACCACAAA-3'
4829	Gst-d1	5'-ACTCCGACTACTACTTCCC-3'/5'-AACCCTGCCAGTTCTCAT-3'
5789	Gst	5'-CCCACATTACCTGACGAT-3'/5'-TACAAAGCACCAACCACC-3'
5907	Gst-e7	5'-TAAAGTTCCGAAATGGGTG-3'/5'-AAGGCGATAATGGCGTGA-3'
1910	SOD	5'-ACGGTGATGTCAAGGGTA-3'/5'-CACGATGCTATTTGCTCC-3'
3139	POD	5'-CGTGAACATCGCATCG-3'/5'-TCGGTTGTGGGAGCAT-3'
5415	AOD	5'-AGGGTCCAATGAAGGGTA-3'/5'-CGATTAAATGCTGCTGA-3'
5659	POD	5'-ATTTGACTTTGATGGAGGTA-3'/5'-GACGGATCTTTGAATGAGCT-3'
3260	Est	5'-GTATGGCAAAGTGAAAGG-3'/5'-GTGAGGTGAGCGTAAGAA-3'
4708	Est	5'-ACATATCTGTACCGCTTCG-3'/5'-ATTTGGGTTACCAGTTGC-3'
5644	P450	5'-TGAAATACCAGCAATCGT-3'/5'-AAAGTAGACACCGCAGAA-3'

Table S4. Growth rate of resistance of B. dorsalis adults to after selection with different time interval

Selection time interval (d)	BR	RT	GF	GR
30	12.961	20.192	7.231	1.558
60	12.961	16.250	3.289	1.254
90	12.961	11.130	-1.831	0.859
120	12.961	10.394	-2.567	0.802
270	12.961	8.738	-4.223	0.674

Abbreviations: BR, Basal resistance ratio; RT, resistance ratio after treatment; GF, Growth times of resistance; GR, Growth rate of resistance.

Table S5. Sequence composition table of SSH library contigs from oriental fruit fly resistant strains

Congtig size (bp)	Number of contigs	
	Trichlorphon strain	Alphamethrin strain
1	103	124
2-5	16	17
6-10	13	7
22-59	4	4
Total	136	152

Table S8. Differentially expressed genes in resistant strains of oriental fruit fly RT-PCR

No. /gene	Relative expression			No. /gene	Relative expression		
	$Tri\text{-}R_1$	$Tri\text{-}R_2$	$Tri\text{-}R_3$		$Alp\text{-}R_1$	$Alp\text{-}R_2$	$Alp\text{-}R_3$
Tri-R2-H9/ cyp6a23	1.19069	7.89542	22.99985	Alp-R3-32/ cyp6g1	0.8390	3.1276	18.2236

Tri-R2-B10/ cyp6g2	1.4179	3.29207	8.11463	Alp-R3-151/ cyp6g1	1.8738	2.0398	11.7810
Tri-R2-382/ JHI	0.3297	1.9129	3.42699	Alp-R3-220/ cyp6y4	1.0771	3.1063	10.4126
Tri-R2-401/ EF1-gamma	1.36675	2.5257	5.1721	Alp-R3-321/ cyp6a17	1.3158	2.3140	3.6169
Tri-R3-D8/ cy8453	1.31581	2.31405	3.61692	Alp-R2-F12/ p450	1.4888	1.8342	2.1785
Tri-R3-116/ GST	1.15177	4.68609	9.90282				
Tri-R3-304/ cyp6g1	1.80866	4.03734	6.84572				

Table S9. Relative expression levels of resistance-related genes in insecticide-resistant strains of various types

Gene	Expression		Gene	Expression	
	Tri-R_3	Alp-R_3		Tri-R_3	Alp-R_3
Tri-R2-A9/ P450	9.5389	17.69829	Alp-R3-151/ P450	0.96771	11.7810
Tri-R2-382/ JHI	3.4269	1.25706	Alp-R3-175/ P450	12.71557	14.2552
Tri-R2-B10/ P450	8.1146	12.20562	Alp-R3-220/ P450	7.32315	10.4126
Tri-R3-D8/ P450	3.6169	6.77658	Alp-R3-231/ P450	8.34411	6.3958
Tri-R3-40/ P450	10.0389	19.91062	Alp-R3-321/ P450	17.58054	3.6169
Tri-R3-116/ GST	9.9028	20.09974	Alp-R2-F12/ P450	1.51354	2.1785

Table S10. The resistance target gene of the microarray

Differentially expressed gene sources	unigenes	Detected gene
Trichlorfon strain	132	81
Alphamethrin strain	156	63
Total	288	144

Table S6. The classification of molecular function from SSH library of resistant to trichlorphon

SeqName	Hit-Desc	Fuction
Contig_78	ubiquitin-like domain-containing ctd phosphatase 1	phosphoprotein phosphatase activity
Contig_56	suppressor of fused	transcription factor binding
Contig_53	stearoyl-coa desaturase	stearoyl-CoA 9-desaturase activity
Contig_106	solute carrier family 12 member 9	sodium:potassium:chloride symporter activity
Contig_92	signal-transducer and activator of transcription protein at isoform b	protein binding; RNA polymerase II core promoter; proximal region sequence-specific DNA binding transcription factor activity involved in positive regulation of transcription
Contig_5	rps6-p70-protein partial	ribosomal protein S6 kinase activity; ATP binding
Contig_93	ring finger and spry domain-containing protein 1	zinc ion binding
Contig_62	ribosomal protein s29	metal ion binding; structural constituent of ribosome RNA binding
Contig_10	retrovirus-related pol polyprotein from transposon tnt 1-94	binding
Contig_89	pyrroline-5-carboxylate dehydrogenase	1-pyrroline-5-carboxylate dehydrogenase activity
Contig_72	pumilio domain-containing protein c14orf21-like	RNA binding
Contig_9	polyprotein	helicase activity; RNA binding; nucleotidyltransferase activity; cysteine-type peptidase activity; nucleotide binding
Contig_74	polyprotein	hydrolase activity
Contig_59	polyprotein	hydrolase activity
Contig_4	polyprotein	hydrolase activity
Contig_32	polyprotein	hydrolase activity
Contig_19	polyprotein	hydrolase activity
Contig_8	nonstructural polyprotein	peptidase activity; binding; transferase activity
Contig_143	nonstructural polyprotein	binding ； hydrolase activity
Contig_144	nonstructural polyprotein	binding; hydrolase activity
Contig_3	nonstructural polyprotein	hydrolase activity
Contig_142	nonstructural polyprotein	hydrolase activity
Contig_139	nonstructural polyprotein	hydrolase activity
Contig_11	nonstructural polyprotein	hydrolase activity

Contig_137	non-structural polyprotein	hydrolase activity
Contig_83	nitric oxide synthase	flavin adenine dinucleotide binding; zinc ion binding; nitric-oxide synthase activity; NADP binding;heme binding; RNA binding; calmodulin binding; FMN binding
Contig_127	neprilysin isoform b	endopeptidase activity
Contig_57	negative elongation factor b	ranslation elongation factor activity
Contig_124	multi drug resistance 50	drug transmembrane transporter activity; ATPase activity, coupled; nucleotide binding
Contig_48	maltase a1	alpha-glucosidase activity
Contig_112	lysosomal enzyme receptor	protein binding
Contig_91	low quality protein: partial	selenium binding
Contig_99	low quality protein: e3 ubiquitin-protein ligase huwe1-like	ligase activity
Contig_60	late trypsin	serine-type endopeptidase activity
Contig_113	juvenile hormone esterase	carboxylesterase activity
Contig_110	isoform n	actin binding;structural constituent of cytoskeleton
Contig_64	isoform d	D-amino acid transmembrane transporter activity; neutral amino acid transmembrane transporter activity; sodium:amino acid symporter activity; neurotransmitter:sodium symporter activity
Contig_38	isoform c	zinc ion binding; transmembrane signaling receptor activity; protein binding; chromatin binding; calcium ion binding
Contig_44	isoform c	Notch binding; repressing transcription factor binding; high mobility group box 1 binding; RNA polymerase II distal enhancer sequence-specific DNA binding transcription factor activity; RNA polymerase II core pr omoter proximal region sequence-specific DNA binding transcription factor activity involved in positive regulation of transcription; RNA polymerase II regulatory region sequence-specific DNA binding
Contig_140	isoform b	zinc ion binding
Contig_123	isoform a	glycogen (starch) synthase activity
Contig_104	hemomucin	strictosidine synthase activity
Contig_126	glutathione s-transferase	transferase activity
Contig_80	elongation factor 2	translation elongation factor activity; GTPase activity; GTP binding
Contig_20	elongation factor 1-gamma	translation elongation factor activity

Contig_90	cytochrome p450-4p1	oxidoreductase activity; iron ion binding; electron carrier activity
Contig_150	cytochrome p450-4p1	metal ion binding; oxidoreductase activity
Contig_66	cytochrome p450	oxidoreductase activity; iron ion binding
Contig_71	cytochrome p450	heme binding;oxidoreductase activity, acting on paired donors, with incorporation or reduction of molecular oxygen monooxygenase activity;electron carrier activity
Contig_125	cytochrome p450	metal ion binding;oxidoreductase activity
Contig_1	cyp6g1	oxidoreductase activity;iron ion binding
Contig_2	cyp6g1	iron ion binding
Contig_36	cytochrome p450 =cypvig2	metal ion binding;oxidoreductase activity
Contig_63	chondroitin sulfate synthase 1	glucuronosyl-N-acetylgalactosaminyl-proteoglycan; 4-beta-N-acetylgalactosaminyltransferase activity
Contig_153	cytochrome p450 =cg8453	electron carrier activity;monooxygenase activity; heme binding;oxidoreductase activity, acting on paired donors, with incorporation or reduction of molecular oxygen
Contig_151	cytochrome p450 =cg8453	oxidoreductase activity;electron carrier activity;iron ion binding
Contig_12	carbonic anhydrase 1	zinc ion binding;carbonate dehydratase activity
Contig_87	cytochrome p450 6a23 =cypvia23	oxidoreductase activity;iron ion binding;electron carrier activity
Contig_34	cytochrome p450 6a23 =cypvia23	oxidoreductase activity;iron ion binding;electron carrier activity
Contig_54	cytochrome p450	oxidoreductase activity;iron ion binding
Contig_120	aldo-keto reductase	alditol:NADP+ 1-oxidoreductase activity
Contig_42	acyl- oxidase	flavin adenine dinucleotide binding;pristanoyl-CoA oxidase activity;acyl-CoA dehydrogenase activity
Contig_103	GJ23986	lipase activity;transporter activity

Table S7. The classification of molecular function from SSH library of resistant to alphamethrin

SeqName	Hit-Desc	Fuction
Contig_173	150 kda dynein-associated polypeptide	microtubule motor activity;myosin II binding;RNA polymerase II distal enhancer sequence-specific DNA binding transcription factor activity;zinc ion binding;nucleic acid binding;dynein binding;
Contig_13	AGAP008582-PA	alpha-mannosidase activity;carbohydrate binding; zinc ion binding
Contig_89	GI24316	metalloendopeptidase activity

Contig_78	GL20838	serine-type carboxypeptidase activity
Contig_92	adult cuticle protein 1	structural constituent of adult chitin-based cuticle
Contig_147	probable cytochrome p450 =cypvia21	metal ion binding
Contig_45	cg33178	glutathione transferase activity
Contig_16	cg5915-pa	receptor activity;GTPase activity;GTP binding
Contig_17	cytochrome p450 = cg8453	metal ion binding;oxidoreductase activity;electron carrier activity
Contig_72	chromosome region maintenance protein 1 exportin	protein binding;nuclear export signal receptor activity
Contig_2	cyp6g1	metal ion binding;oxidoreductase activity
Contig_26	cyp6g1	oxidoreductase activity;iron ion binding
Contig_31	cyp6g1	oxidoreductase activity;iron ion binding
Contig_139	cyp6g1	oxidoreductase activity;iron ion binding
Contig_142	cyp6g1	oxidoreductase activity;iron ion binding
Contig_150	cyp6g1	oxidoreductase activity;iron ion binding
Contig_149	cyp6g1	heme binding;oxidoreductase activity, acting on paired donors, with incorporation or reduction of molecular oxygen;electron carrier activity
Contig_41	cytochrome oxidase subunit partial	Electron carrier activity; heme binding;cytochrome-c oxidase activity
Contig_56	cytochrome p450	electron carrier activity;monooxygenase activity; heme binding;oxidoreductase activity, acting on paired donors, with incorporation or reduction of molecular
Contig_61	cytochrome p450	heme binding;oxidoreductase activity, acting on paired donors, with incorporation or reduction of molecular oxygen;monooxygenase activity;electron carrier activity
Contig_67	cytochrome p450 partial	heme binding;oxidoreductase activity, acting on paired donors, with incorporation or reduction of molecular oxygen;monooxygenase activity;electron carrier activity
Contig_37	cytochrome p450-6a17	heme binding;oxidoreductase activity, acting on paired donors, with incorporation or reduction of molecular oxygen;monooxygenase activity; electron carrier activity
Contig_25	cytochrome p450-6g4	oxidoreductase activity;iron ion binding;electron carrier activity
Contig_77	epoxy hydrolase	epoxide hydrolase activity;cis-stilbene-oxide hydrolase activity

Contig_119	genghis khan	small GTPase regulator activity;protein serine/threonine kinase activity;metal ion binding;phospholipid binding; ATP binding; diacylglycerol binding
Contig_109	guanylatte cyclase alpha 1 subunit	guanylate cyclase activity;nucleotide binding; heme binding
Contig_33	imaginal disc growth factor 1	chitinase activitycation binding;imaginal disc growth factor receptor binding
Contig_118	isoform a	protein tyrosine phosphatase activitytransferase activity;phospholipid binding;Rho guanyl-nucleotide exchange factor activity
Contig_35	isoform b	UDP-glycosyltransferase activity;transferase activity, transferring hexosyl groups
Contig_52	isoform b	protein tyrosine/serine/threonine phosphatase activity
Contig_57	isoform b	heat shock protein binding
Contig_70	isoform b	zinc ion binding;microtubule-severing ATPase activity;metalloendopeptidase activity;ATP binding
Contig_98	isoform c	calcium ion binding
Contig_106	isoform c	structural constituent of muscle;motor activity
Contig_48	isoform d	calcium ion binding;tropomyosin binding
Contig_105	isoform d	long-chain fatty acid transporter activity;oxidoreductase activity
Contig_36	isoform f	acid phosphatase activity
Contig_58	maltase a1	alpha-glucosidase activity;cation binding
Contig_21	mind bomb isoform a	myosin II light chain bindingzinc ion binding; oxidoreductase activity;ubiquitin-protein ligase activity;myosin II heavy chain binding
Contig_116	nucleolar protein 6-like	RNA binding
Contig_51	pancreatic triacylglycerol lipase	hydrolase activity
Contig_15	polyprotein	hydrolase activity
Contig_49	polyprotein	Binding;hydrolase activity
Contig_81	polyprotein	hydrolase activity
Contig_152	polyprotein	hydrolase activity
Contig_115	replication protein a 70	single-stranded DNA binding
Contig_121	rigor mortis	protein binding

Contig_42	rna-binding protein isoform b	mRNA 3'-UTR binding;nucleotide binding
Contig_12	rrna promoter binding protein	receptor activity;monooxygenase activity
Contig_6	serine protease	peptidase activity
Contig_30	succinyl- synthetase small	ATP citrate synthase activity;rRNA binding;structural constituent of ribosome;succinate-CoA ligase (ADP-forming) activity;nucleotide binding; succinate-CoA ligase (GDP-forming) activity;cofactor binding;
Contig_5	synaptic glycoprotein sc2	oxidoreductase activity, acting on the CH-CH group of donors
Contig_93	trna selenocysteine 1-associated protein 1-like	nucleic acid binding;nucleotide binding
Contig_1	yolk protein 1	catalytic activity;structural molecule activity
Contig_157	yolk protein 1	catalytic activity
Contig_159	yolk protein 1	catalytic activity
Contig_174	yolk protein 1	catalytic activity
Contig_27	yolk protein 2	catalytic activity
Contig_34	yolk protein 2	catalytic activity;structural molecule activity
Contig_161	yolk protein 2	catalytic activity;structural molecule activity
Contig_4	yolk protein 3	catalytic activity
Contig_160	yolk protein 3	structural molecule activity
Contig_167	yolk protein 3	catalytic activity;structural molecule activity
Contig_168	yolk protein 3	catalytic activity;structural molecule activity
Contig_166	yolk protein a	catalytic activity
